# *MoveMentor*—examining the effectiveness of a machine learning and app-based digital assistant to increase physical activity in adults: protocol for a randomised controlled trial

**DOI:** 10.1186/s13063-025-08926-3

**Published:** 2025-07-01

**Authors:** Corneel Vandelanotte, Stewart Trost, Danya Hodgetts, Tasadduq Imam, MD Mamunur Rashid, Quyen G. To, Carol Maher

**Affiliations:** 1 Appleton Institute, Central Queensland University, Bruce Highway, Rockhampton, QLD 4702 Australia; 2 School of Human Movement and Nutrition Science, The University of Queensland, St Lucia, QLD 4072 Australia; 3 School of Business and Law, Central Queensland University, 120 Spencer Street, Melbourne, VIC 3000 Australia; 4 School of Engineering and Technology, Central Queensland University, 120 Spencer Street, Melbourne, VIC 3000 Australia; 5 Alliance for Research in Exercise, Nutrition and Activity, Allied Health and Human Performance, University of South Australia, City East Campus, Adelaide, SA 5001 Australia

**Keywords:** Chatbot, Conversational agent, Exercise, Steps, Artificial intelligence, Reinforcement learning, Large language model, Natural language processing, Behaviour change, Intervention, Just-in-time adaptive intervention, Mhealth, Digital, Personalised, Customised

## Abstract

**Background:**

Physical inactivity is prevalent, leading to a high burden of disease and large healthcare costs. Thus, there is a need for affordable, effective and scalable interventions. However, interventions that are affordable and scalable are beset with modest effects and engagement. Interventions that integrate machine learning with real-time data to offer unprecedented levels of personalisation and customisation might offer solutions. The aim of this study is to conduct a randomised controlled trial to evaluate the effectiveness of a machine learning and app-based digital assistant to increase physical activity.

**Methods:**

One hundred and ninety-eight participants will be recruited through Facebook advertisements and randomly allocated to an intervention or control group. Intervention participants will gain access to an app-based physical activity digital assistant that can learn and adapt in real-time to achieve high levels of personalisation and user engagement by virtue of applying a range of machine learning techniques (i.e. reinforcement learning, natural language processing and large language models). The digital assistant will interact with participants in 3 main ways: (1) educational conversations about physical activity; (2) just-in-time personalised in-app notifications (‘nudges’), cues to action encouraging physical activity and (3) chat-based questions and answers about physical activity. Additionally, the app includes adaptive goal setting and an action planning tool. The control group will gain access to the intervention after the last assessment. Outcomes will be measured at baseline, 3 and 6 months. The primary outcome is device-measured (Axivity AX3) moderate-to-vigorous physical activity. Secondary outcomes include app engagement and retention, quality of life, depression, anxiety, stress, sitting time, sleep, workplace productivity, absenteeism, presenteeism and habit strength.

**Discussion:**

The trial presents a unique opportunity to study the effectiveness of a new generation of digital interventions that use advanced machine learning methods to improve physical activity behaviour. By addressing the limitations of existing conversational agents, we aim to pave the way for more effective and adaptable interventions.

**Trial registration:**

Australian New Zealand Clinical Trial Registry ACTRN12624000255583p. Registered on 14 March 2024. https://www.anzctr.org.au/Trial/Registration/TrialReview.aspx?id=387332.

**Supplementary Information:**

The online version contains supplementary material available at 10.1186/s13063-025-08926-3.

## Introduction

There is a wealth of evidence regarding the benefits of regular physical activity [1]. Physical activity guidelines recommend a minimum of 150 min of moderate-to-vigorous physical activity per week to prevent the development of cardiovascular disease, diabetes, obesity, cancer and depression [2, 3]. Yet, more than a third of the world’s population does not meet these guidelines [4], causing 9.4% of the total burden of disease and estimated to cost INT$520 billion between 2020 and 2030 through healthcare costs and lost productivity [5, 6]. Therefore, there is an important need to develop and evaluate interventions that can increase physical activity at a population level and at an affordable cost.

Unfortunately, increasing physical activity at a population level has been difficult to achieve. While intensive face-to-face counselling by health professionals is effective, it cannot be implemented at scale due to limited feasibility and high costs [7]. Alternative methods to increasing activity levels at scale (e.g. phone, print, websites, apps) have shown promise, but effect sizes tend to be modest [8–10]. As such, the challenge to develop effective, affordable and scalable physical activity interventions remains. In this context, the application of machine learning and artificial intelligence to increase activity levels is increasingly gaining interests by behavioural scientists.

While the concepts of machine learning and artificial intelligence were first established in the 1950 s [11], it has taken more than 60 years for their broad application and implementation in today’s society, due to a lack of computer processing power and speed [12]. Though, right now, we are experiencing nothing short of a revolution in terms of the rapid proliferation and adoption of very powerful machine learning applications (e.g. it took less than 2 months for ChatGTP’s large language model to reach a 100 million users).

Previously, computer-based expert systems have applied basic If–Then decision-rules to provide personal physical activity advice (so-called computer-tailored interventions) [13]. Though these interventions demonstrated superior effectiveness compared to generic or targeted interventions [14], they are not optimally suited to handle the complex and dynamic nature of behaviour change and are not able to provide the right support at the right time (i.e. just-in-time adaptive interventions) [15]. Yet, physical activity interventions that apply machine learning algorithms might be able to overcome the limitations of the previous generation of computer-based interventions.

Some have already examined the use of machine learning to increase physical activity levels [16–18], and while the outcomes of these studies have been promising, it is important to point out that the methodological rigour in those studies was low and that the true application of machine learning has been limited. Most of these studies have examined chatbots that have applied off the shelf natural language processing (NLP) but have not implemented machine learning techniques that involved high levels of personalisation or the use of real-time data from activity sensors to deliver just-in-time messages [16–18].

Therefore, based on our initial work in this area [19, 20], we published an innovative concept of a sophisticated machine learning-based digital assistant, able to provide highly personalised physical activity advice, delivered just-in-time based on real-time data [21]. After completing focus group research [22], co-creation workshops, software development and extensive beta-resting, we have now developed a digital assistant that is able to dynamically and proactively nudge users into being more physically active. Our intervention applies machine learning techniques rarely applied to increase physical activity to date (i.e. reinforcement learning). In addition, the intervention also uses NLP, along with an integrated large language model (i.e. Google’s Gemini) that can formulate coherent and relevant answers to any physical activity-related questions participants may have. Now that the development phase has been completed, we are ready to evaluate the effectiveness of this new intervention. For this evaluation, we will focus on workplace-based employees, as workplaces offer a captive audience to implement interventions to large populations for potential future translation efforts [23]. Moreover, workplace interventions can achieve outcomes that go beyond health benefits, such as improved productivity, workplace morale, reputation and lower absenteeism [24]. Therefore, the aim of this study is to conduct a randomised controlled trial to examine the effectiveness of a machine learning and app-based digital assistant to increase physical activity in office-based employees. We hypothesise that participants receiving the intervention will significantly increase their moderate to vigorous physical activity at 3- and 6-month follow-ups compared to participants allocated to a no intervention control group.

## Methods

### Trial design

The MoveMentor trial is a parallel group, two-arm, superiority trial with a 1:1 allocation ratio to investigate the effectiveness of an app-based digital assistant to increase physical activity compared to a no intervention control group. Given the novelty of the intervention, we first need to establish the effectiveness of the intervention against a true control group, prior to comparing it to alternative interventions. The control group will be offered access to the intervention after the final assessment has been completed. The study setting is ‘home-based’, as there will be no face-to-face contact with participants, and all communication will occur via e-mail or phone. Primary and secondary outcomes will be assessed at baseline, 3 months and 6 months. The study has received ethics approval from the Human Research Ethics Committee (HREC) of the Central Queensland University (0000024786) and complies with the Helsinki Declaration. While not anticipated, any adverse events will be reported immediately to the HREC and managed according to their instructions and reported in publications resulting from this study. Likewise, ethical approval will be sought prior to any protocol amendments should they be needed. The Central Queensland University is the sponsor for this trial. There are no plans to use participants’ data in any other studies. This study protocol has been prepared in accordance with SPIRIT guidelines (see Additional file 1 and Table 1) and study outcomes will be reported according to CONSORT guidelines [25, 26].

**Table 1:**
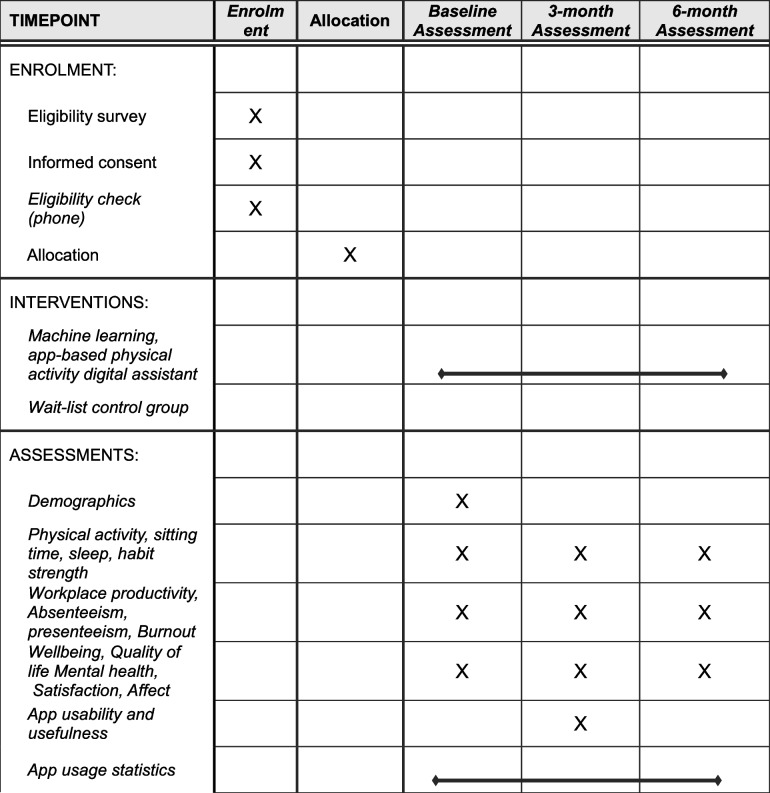
SPIRIT figure

### Participants

One hundred and ninety-eight participants will be recruited through Facebook advertisements. As response rates to Facebook advertisements are lower in men [27], more funds will be spent on advertisements targeting men, as to obtain a reasonably balanced sample. Recruitment rates will be closely tracked throughout the recruitment phase to determine how much should be spent on advertisements for each gender. All advertisements will direct interested individuals to a Qualtrics survey for screening. After potential participants have completed the screening questionnaire and are deemed eligible, they will be provided with a detailed study information sheet. If those deemed eligible are still interested in participating, they will be asked to provide contact details and informed consent. Those deemed ineligible will be informed of why, but they will not be able to continue.

Eligible participants will be:

- Over 18 years of age
- Live anywhere in Australia
- Speak and read the English language
- Engaged in full-time office-based employment
- Have a smartphone (i.e. Apple or Android) with internet access
- Be physically inactive. Participants will be asked: ‘*How many days per week do you engage in at least 30 min of activity of a moderate intensity or physical activity higher? For example, brisk walking, swimming, tennis, *etc.*?*’ Anyone reporting more than 2 days per week will be excluded.
- Have no impairments limiting an increase in physical activity
- Did not participate in any physical activity programs in the past 12 months
- Not own or have used a physical activity tracker for at least 12 months
- Not be pregnant
- Have a body mass index over 17.5

### Procedure

When contact details of eligible people are received through the Qualtrics survey, a project officer will make contact by phone to verify the information provided, make sure they are aware of and understand all study requirements and that they still agree to participate. Subsequently, they will be e-mailed a link to access to an online baseline survey (administered via Qualtrics). Participants will also be sent an accelerometer-based activity monitor using registered mail, as well as instructions on how to use the device, a 7-day monitoring log and a pre-paid return post bag. They will be asked to wear the activity monitor on the non-dominant wrist for 7 consecutive days and nights from the day of receipt. When the accelerometer is returned to the laboratory, it will be checked for valid wear time. Potential participants providing less than four valid monitoring days (≥ 10 h of wear time) will be excluded from the study. The same measurement procedures (online survey and 7-day accelerometer) will be repeated at 3 and 6 months (though participants with incomplete data will not be excluded at this stage). Participants will be rewarded with a $50 AUD shopping voucher for each completed assessment. Participants can withdraw from the study without any consequence at any stage. Whenever needed, reminder e-mails and phone calls will be used to encourage participants to complete assessments.

In accordance with the approved study data management plan (DMP_2790), data from all sources will be securely stored for a minimum of 10 years on a university server dedicated to research data and regularly backed up. Only study investigators named on this publication will have access to the final trial dataset. Given the small scale of this study, no data monitoring committee will be established beyond the involvement of the study investigators named on this publication. Participants’ identity will be protected at all times; only de-identified and aggregated data will be disseminated in publications and reports. A plain English statement summarising trial outcomes will be shared with participants that indicated an interest. Publications resulting from this study will abide by ICMJE authorship guidelines and not involve professional writers.

**Fig. 1:**
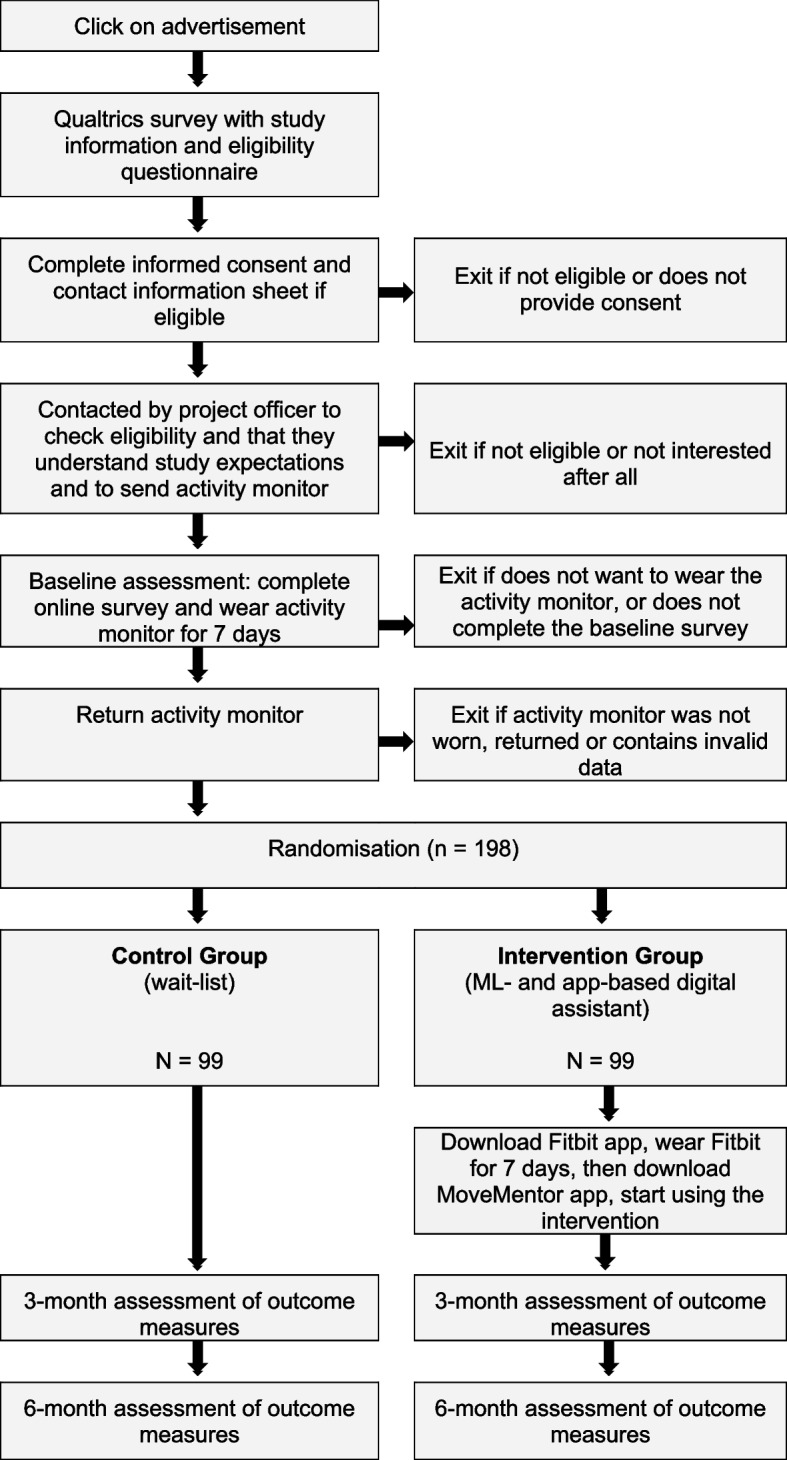
Flowchart

When the initial activity monitoring period and baseline survey have been completed, participants will be randomised into one of two groups using a randomly generated sequence created by CV within Microsoft Excel (version 16.89.1, Microsoft Corporation, Redmond, WA). The column containing the randomised sequence will be concealed in the spreadsheet and will only be unmasked row-by-row by the study manager (DH) after a new participant has been added to the spreadsheet. After randomisation, intervention group participants will be sent a Fitbit Inspire 2 activity tracker (Fitbit Inc, San Francisco, CA) as part of the intervention. They will be asked to download the Fitbit app onto their smartphone, connect their Fitbit tracker with this app and wear the Fitbit for at least 7 days. The Fitbit is worn on a participant’s preferred wrist, with the dominant or non-dominant wrist preference recorded in the Fitbit app on setup. After this, they will be asked to download the digital assistant smartphone app (i.e. *MoveMentor*) onto their smartphone and grant permission for *MoveMentor* to access their Fitbit data. Once everything is set up, intervention group participants will be asked to wear their Fitbit as much as possible and actively engage with the *MoveMentor* app during the 6-month intervention period. Intervention participants’ use of *MoveMentor* will be monitored for the entire 6-month study duration. Intervention participants who do not download or use *MoveMentor* will be encouraged to download and/or use to use the app via e-mail. Given the behavioural nature of the study, aspects of concomitant care are not applicable in this study. At the end of the study, intervention group participants will be able to keep the Fitbit activity tracker and access to the MoveMentor app will continue to be available via the Apple App and Google Play stores for all study participants. The flowchart in Fig. 1 shows an overview of the study procedures.

### Intervention

**Fig. 2:**
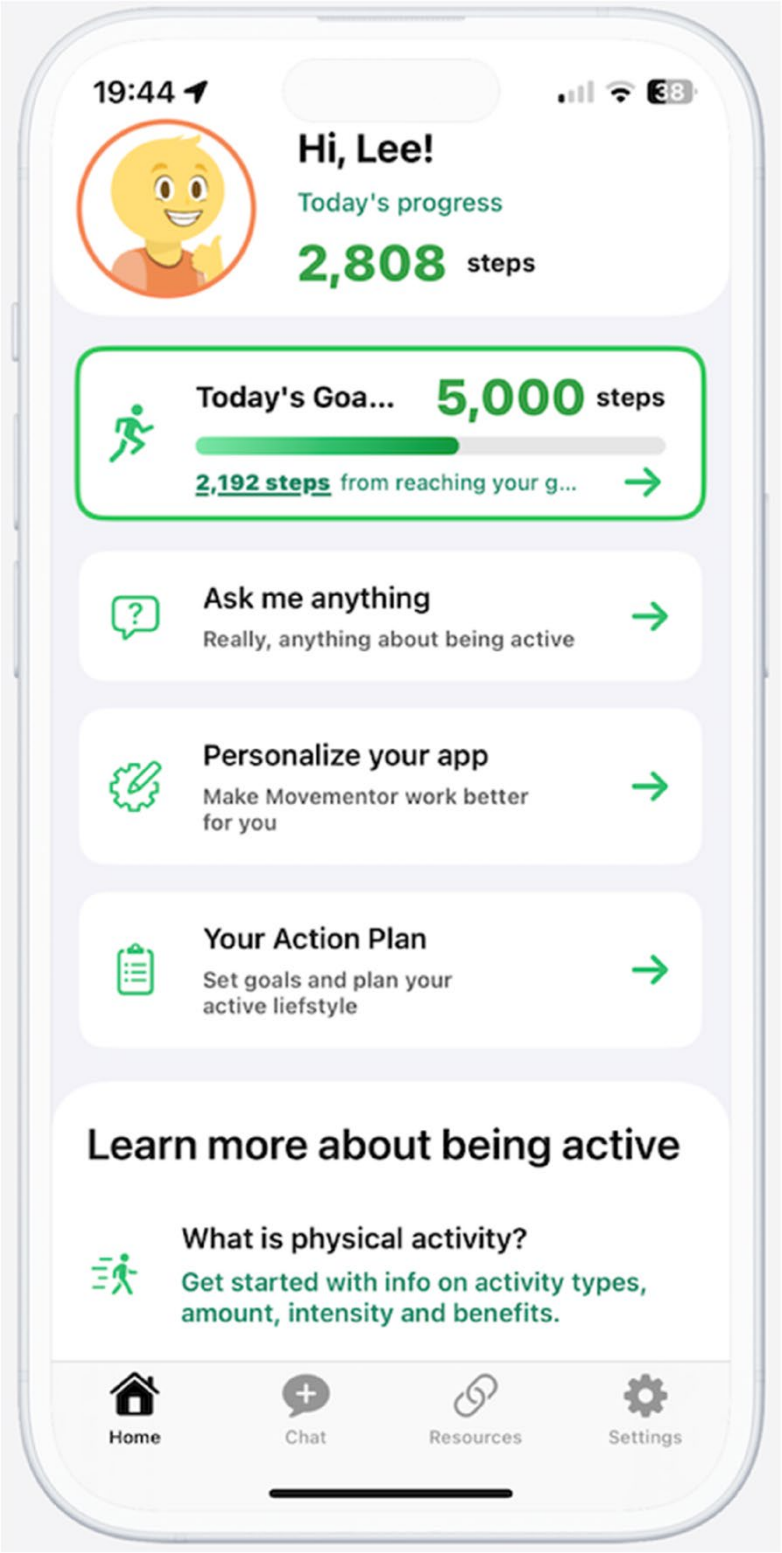
Image of the MoveMentor app homepage

A detailed description of the intervention concept has been published elsewhere [21]; this includes in-depth information on how machine learning aspects have been applied. Briefly, the intervention consists of a stand-alone smartphone app (for both Apple and Android operating systems) named *MoveMentor* (see Fig. 2). The app works in conjunction with physical activity trackers (e.g. Fitbit). The main feature of the app is the physical activity digital assistant (i.e. chatbot or conversational agent). While the name of the digital assistant in the app stores is *MoveMentor*, users can change the name inside the app and select one of three main characters (i.e. *MoveMentor* (default), *MoveMate* or *MyPT*) or choose their own name and image for the digital assistant.

The digital assistant interacts with participants in 3 main ways: (1) educational conversations; (2) nudges (via smartphone push notifications) and (3) chat-based questions and answers (Q&A). The conversations provide participants with educational content in relation to physical activity delivered through interactive ‘conversations’ or ‘chats’. The aim of the conversations is to encourage participants to become more physically active and meet the national physical activity guidelines (i.e. 150 to 300 min of moderate-to-vigorous intensity physical activity), as well as to support them in overcoming any difficulties in becoming more active [28]. The conversations are supported by Google’s Dialogue Flow and use natural language processing to enable a more natural flow for the conversation. Example conversation topics include ‘*Developing a lasting activity habit*’, ‘*What stops you from being active?*’, ‘*Make the most of your surroundings*’, ‘*Being active with others*’ and ‘*Building confidence for being active*’. During the conversations, the *MoveMentor* will ask participants physical activity questions and participants will be provided with personalised advice, depending on the answers they provide. Additional resources are provided at the end of each conversation for further reading, watching or listening. The conversations are designed to last no longer than 5 or 10 min. A list of conversations is available for participants on the homepage of the app; however, participants will also receive ‘nudges’ (i.e. notifications) to engage in these conversations. Participants will be able to go through a conversation more than once if they want to do so. The conversations have been informed by the Self-Determination and Social Cognitive Theories [29, 30].

Based on the ‘Nudge’ and ‘COM-B’ theories (Capability-Opportunity-Motivation and Behaviour), participants will also be nudged into action [31, 32]. The nudges (delivered as a smartphone push notification) should be thought of as a Just-In-Time Adaptive Intervention (JITAI), which uses real-time data to dynamically address user needs by providing the right cue to action at the right time to assist with long-term habit formation [15]. The timing and content of the nudges are determined by machine learning algorithms that learn what works for whom under what circumstances through trial and error. The machine learning algorithm is trained to make the nudges more personally relevant over time and will use real-time feedback from activity trackers to learn what nudges work and do not work (i.e. an increase in physical activity following nudges or not). The nudges that work will be reinforced, and those that do not will be phased out. More specifically, a ‘contextual bandit’ approach is applied to deliver the right nudges at the right time; this is a type of reinforcement learning [33, 34]. Contextual data will be derived from participants’ app preferences and settings (derived using an onboarding survey), participant interactions with the app and digital assistant, physical activity data from the activity trackers, nudge ratings (i.e. like or dislike) and weather data (to determine whether indoor or outdoor activity is recommended based on the participants’ location at that specific time). An extensive library of nudges has been developed to send to users through push notifications. The library consists of a range of nudge categories from which nudges are selected in a random pattern. However, a nudge that has been used before will not be used again unless all other nudges within a category have been used as well. Examples of nudge categories are ‘daily morning check-in’, ‘weekly action plan review’, ‘activity suggestion’ (e.g. inspirational, based on known participant characteristics, based on weather), ‘motivational’ (i.e. to encourage meeting their daily activity goal), ‘educational’ (i.e. physical activity facts, tips and tricks), ‘streaks’ (i.e. achieving daily goals repeatedly), ‘milestones’ (e.g. recording over a million steps) and more. The reinforcement learning algorithms have been trained with the help of 300 beta-testers over a 6-month period to improve their performance.

Finally, participants can engage with the digital assistant by asking it physical activity-related questions. Answers to questions that have not been pre-programmed (e.g. I have a muscle pain in my calf, how can I still be active?) are being delivered by Gemini (Alphabet Inc, Mountain View, CA), Google’s machine learning and natural language generation model (it is a large language model comparable to ChatGPT). To generate the best answer, additional contextual information will be sent to Gemini. This will include previous conversations, the user’s nominated username, recent physical activity, local weather and other information provided during the usage of the app. The answer will be generated by the Gemini algorithms.

Beyond interactions with the digital assistant and personalisation features, the *MoveMentor* app includes additional features, of which adaptive goal setting and action planning are the most important. Every morning, participants receive a daily activity goal (expressed as steps or activity minutes, as per participant preference). This goal is based on whether they met their activity goal during the previous day, as well as the average physical activity conducted in the last 4 weeks. The goal is designed to slowly increase over time, aligned with how well participants are performing, until it reaches a participant’s ‘long-term activity’ goal (defined as part of the action planning process), at which point the goal stops increasing. If participants are consistently not meeting their daily activity goal, then the goal will gradually decrease, to better align with the participant’s activity journey.

Participants are encouraged to engage in an action planning process [35]. This conversation is designed to help participants formulate a weekly action plan. If participants are to achieve their long-term goal, they need concrete short-term action. As such, the action plan helps participants define what physical activities they will engage in, where they will do them, how many times per week, what days of the week, what time of day, how long activity sessions will last and identify any people they may be active together with. To be effective, action plans need to be updated regularly and the digital assistant will send out a weekly reminder to review participants’ action plans.

Control group participants will not receive access to the intervention until after the 6-month waiting period. They will not receive a Fitbit activity monitor.

### Measures

#### Primary outcome

Device-measured moderate-to-vigorous physical activity: Participants will be asked to wear an Axivity AX3 tri-axial accelerometer (https://axivity.com/product/ax3) on their non-dominant wrist for 7 consecutive days during waking and sleeping hours, except when swimming, bathing or participating in contact sports. Participants will also be asked to complete an activity monitor log detailing times the monitor was removed, time they went to bed and wake up time. The Axivity AX3 has demonstrated good concurrent validity [36].

Raw accelerometer data collected at 50 Hz will be processed into physical activity metrics using a machine-learned random forest classifier specifically designed and validated for assessing movement behaviours in free living adults [37]. The random forest classification model uses features in the raw acceleration signal to classify each 10-s window (epoch) as sedentary (lying or sitting still), stationary plus (active sitting, standing still, active standing), walking or running. Daily time spent in moderate-to-vigorous intensity physical activity (MVPA) will be calculated using the approach established by Ahmadi et al. in their analysis of accelerometer data from the UK biobank study [38]. Windows classified as walking with normalised gravitation units (milli g) ≥ 100 milli g and < 400 milli g will be considered moderate intensity physical activity (3–6 METs). Windows classified as walking with normalised gravitational units ≥ 400 milli g will be considered vigorous intensity physical activity (≥ 6 METs), while all windows classified as running will be considered vigorous intensity physical activity (≥ 6 METs). Non-wear periods will be identified and differentiated from sleep using the methods described by Ahmadi and colleagues [39]. The participants’ accelerometer data will be included in the analyses if they have ≥ 4 days (with at least 1 weekend day) in which daily wear time is ≥ 10 h [40]. Device-measured physical activity will be reported as the average minutes of MVPA per day or week for each time point and group.

#### Secondary outcomes

##### Health behaviours

- Device-measured sedentary time and sleep will also be assessed using the Axivity AX3 tri-axial accelerometer. Sleep periods will be detected using the Van Hees et al. heuristic [41].
- Self-reported physical activity will also be assessed. The Active Australia Survey provides contextual physical activity information as it assesses the frequency and duration (in minutes per week) of walking for transport, walking for recreation, moderate physical activity and vigorous physical activity [42]. The Active Australia Survey has acceptable test–retest reliability and validity in the Australian adult population and has been documented as a useful evaluative tool for detecting intervention related change in physical activity [43, 44].
- Self-reported sitting time will be measured using the 10-item ‘Workforce Sitting Questionnaire’ [45]. All participants will report time (hours/minutes) spent sitting on usual working and non-working days in relation to work, transport, TV use, computer use and other leisure-time sitting. One question also measures the number of days participants usually work in a week. This survey has demonstrated adequate test–retest reliability and validity [45].
- Self-reported sleep behaviour will be assessed using items from both the Behavioural Risk Factor Surveillance Screening Sleep Module [46] and the valid and reliable Pittsburgh Sleep Quality Index [47]. This study assesses average hours of sleep obtained, snoring behaviour, unintentionally falling asleep during the day, nodding off while driving, whether one feels rested and levels of sleep quality. Additionally, an objective measure of sleep will be collected using the Axivity accelerometer. Total sleep time, sleep efficiency, number of awakenings, sleep onset and sleep offset will be extracted from the accelerometer data using established protocols [39].

##### Work-related outcomes

- The Health and Work Questionnaire (HWQ), a survey for assessing workplace productivity, consists of 24 items, with each item rated on a 10-point Likert scale (e.g. ‘very dissatisfied’ to ‘very satisfied’) [48]. The questions assess work quality, quantity and efficiency, as well as impatience, concentration/focus, work satisfaction and non-work satisfaction. The HWQ shows very good internal consistency reliability [48].
- The World Health Organization’s Heath and Work Performance Questionnaire (HPQ), will be used to assess absenteeism and presenteeism [49]. Eight items are used to assess absenteeism and 3 items are used to assess presenteeism. Good concordance was found between the HPQ and other productivity indicators [49].
- The Maslach Burnout Inventory will assess various aspects of burnout syndrome [50]. It has a total of 22 items that are scored on 7-point scales and contains 3 subscales: emotional exhaustion, depersonalisation and personal accomplishment. Various psychometric analyses showed that the scale has both high reliability and validity as a measure of burnout [50].
- The Employee Wellbeing Scale: This survey, with demonstrated validity and reliability [51], assesses employee wellbeing using 18 items on 7-point Likert scales (scored: 1 = strongly disagree; 7 = strongly agree). The survey assesses wellbeing across three dimensions: life wellbeing, work wellbeing and psychological wellbeing.

##### Mental health and quality of life

- Quality of life will be measured through the Short-Form Health Survey (SF-12) which includes questions on physical and mental health. The SF-12 is widely used, valid and reliable [52].
- Depression, anxiety and stress: The 21-item Depression, Anxiety and Stress Scale (DASS21) will be used to assess mental health outcomes [53]. The DASS21 provides a quantitative measure of distress symptom severity; it is not a measure of clinical diagnosis. Three 7-item subscales measure depression, anxiety and stress. These scales have demonstrated acceptable validity and reliability in community samples of adults [53].
- The Satisfaction with Life Scale is a 5-item measure of an individual’s global evaluation of their life [54]. Participants complete the measure on a 1 to 7 Likert-type response scale. Scores can range between 5 and 35, with higher scores indicating greater life satisfaction. The scale has been widely used, with high reliability and validity [55].
- The Positive and Negative Affect Schedule—short form (I-PANAS-SF) is a 10-item Positive and Negative Affect Scale [56]. The scale measures an individual’s experiences of positive and negative affect with two 5-item subscales. Participants complete the measure on a 1 to 5 Likert-type response scale. Scores can range between 5 and 25 for each subscale, with higher scores indicating greater experience of positive or negative emotion. The scale is reliable, valid and has demonstrated cross-cultural validity [57].
- Habit strength will be assessed using the 4-item Self-Reported Behavioural Automaticity Index [58], which is a subscale of the Self-Reported Habit Index [59]. Participants are asked to what extent they agreed with the items, ‘Physical activity is something I do… (1) automatically, (2) without having to consciously remember, (3) without thinking, (4) before I realise I’m doing it’, using response scales from 1 (strongly disagree) to 7 (strongly agree). The habit strength score is calculated as the mean of the 4 items. This scale was found to be reliable and sensitive to the effects of habit on behaviour [58].

##### Intervention use and usability

- App usability and usefulness: The overall usability of the app will be investigated using the System Usability Scale [60]. This 10-item scale has demonstrated acceptable reliability and validity for measuring the usability of websites and apps [60]. Additionally, there will be 5 items on how useful participants found the app in supporting them in becoming more active. These outcomes will only be measured at the 3-month time point.
- App usage statistics: App usage will be measured using the Google Analytics Firebase traffic analysis platform (Alphabet Inc, Mountain View, CA), as well as by monitoring user-generated content. Features monitored will include (but are not limited to) the number of times the app was opened, time in the app, app pages visited, number of action plans completed, number of completed conversations, questions asked in chat, amount of nudge feedback provided, physical activity recorded through the app (i.e. activity minutes and steps) and activity milestones achieved (e.g. times the daily goal was met). App usage will be monitored for the entire study duration.

##### Demographics

The following demographic factors will be assessed: age, sex, weight, height, marital status (single, widowed, divorced, separated, married, in a relationship), ethnicity (Caucasian, Aboriginal or Torres Strait Islander, African, Asian, other), number of years of education, occupational category (e.g. manager, professional, sales), income, postcode, residential environment (e.g. rural area), Accessibility/Remoteness Index of Australia (ARIA) [61] and presence of chronic diseases (e.g. hypertension, stroke, arthritis, depression). Additionally, alcohol consumption will be assessed using the Alcohol Use Disorders Identification Test (AUDIT-C). It is a 3-item test for heavy drinking and/or active alcohol abuse or dependence, with demonstrated validity and reliability [62]. Smoking will be assessed using a single item to classify participants as either a current smoker or non-smoker. Diet will be assessed using four items that evaluated the daily frequency of fruit and vegetable consumption and the number of times soft drinks and fast foods were consumed in the previous 7 days [63]. An overview of all measures and when they are being assessed is provided in Table 2.

**Table 2:** Overview of measures, instruments and when they are being assessed

Measure	Instrument	Timepoints	Groups
In- and exclusion criteria	Eligibility survey (includes informed consent for those eligible)	During recruitment	All those interested
Sample characteristics	Demographic questions [61, 63], AUDIT-C [62]	Baseline	All (i.e. intervention and control groups)
Physical activity	Axivity AX3 accelerometer [36–40]	All (i.e. baseline, 3 months and 6 months)	All
	Active Australia Survey [42–44]	All	All
Sitting time	Axivity AX3 accelerometer [36]		
	Workforce Sitting Questionnaire [45]	All	All
Sleep	Axivity AX3 accelerometer [36, 41]		
	BRFSS Sleep Module [46]	All	All
	Pittsburgh Sleep Quality Index (PSQI) [47]	All	All
Workplace productivity	Health and Work Questionnaire (HWQ) [48]	All	All
Absenteeism and presenteeism	World Health Organization’s Heath and Work Performance Questionnaire (HPQ) [49]	All	All
Burnout	Maslach Burnout Inventory [50]	All	All
Wellbeing	Employee Wellbeing Scale [51]	All	All
Quality of life	Short-Form Health Survey (SF-12) [52]	All	All
Mental health	Depression, Anxiety and Stress Scale (DASS21) [53]	All	All
Satisfaction	Satisfaction with Life Scale [54, 55]	All	All
Affect	Positive and Negative Affect Schedule—short form (I-PANAS-SF) [56, 57]	All	All
Habit strength	Self-Reported Behavioural Automaticity Index (SRBAI) [58, 59]	All	All
App usability and usefulness	System Usability Scale and custom questions [60]	3 months only	Intervention group only
App usage statistics	Google Analytics and app database	Continuous throughout the 6-month intervention period	Intervention group only

### Sample size

Sample size for the study is based on the primary outcome of change in device-measured MVPA (min/day). Several reviews of app-based physical activity interventions suggest that most studies have small to medium effects on change in physical activity and have high dropout rates, up to approximately 30% [64, 65]. Hence, to detect a small to medium difference in the change in daily MVPA between groups (*d* = 0.45; corresponding with a minimal detectable difference of 10 min/day), at the study’s primary time point of 6 months, 76 participants per group will be required to achieve 80% power using an alpha level of 0.05. The number of participants per group will be inflated by 30% (99 participants per group) to account for drop out. Hence, a total of 198 participants will be recruited into the RCT.

### Statistical analyses

Data will be analysed by a statistician blinded to group allocation, using generalised linear mixed models (GLMM), including fixed effects for group, time, the group-by-time interaction and a random subject effect [66]. GLMM analyses examine changes from baseline to each follow-up time point for each intervention group compared to the control group while accounting for within-person nesting of variables across time [66]. Random effects mixed modelling will also be used to explore potential changes in secondary outcomes. Planned subgroup analyses include examining any moderating effects of sociodemographic variables (e.g. sex, age, income) and app usage as potential mediator for increases in physical activity levels. Data will be tested for linearity and normality to ensure the proper statistical techniques are applied. The most appropriate approach to handling missing data will be applied depending on the pattern and amount of the missing data. However, if possible, intention-to-treat including all participants with valid baseline data and multiple imputation by chained equations will be used to handle missing data [67]. The analyses will be conducted using SPSS v30.

## Discussion

The current trial presents a unique opportunity to study the effectiveness of a new generation of digital interventions that use reinforcement learning (i.e. contextual bandit) in combination with real-time data to deliver a highly personalised app-based digital assistant for promoting physical activity [23]. By addressing the limitations of existing conversational agents and applying advanced machine learning techniques, we aim to pave the way for more effective and adaptable interventions. There is an ongoing proliferation of health and physical activity smartphone apps, yet very little work has been carried out to study the utility and effectiveness of these population-targeted apps [68]. Soon, many more of these apps will also be applying various form of machine learning techniques to influence health behaviour. As such, our work will contribute to understanding how effective these approaches are, and what potential downsides might be. We expect that the current study’s findings will provide increased insights into the benefits of applying machine learning in engaging and retaining participants in app-based interventions, something previous interventions have struggled with [69, 70]. Higher participant engagement and retention have been shown to result in higher levels of behaviour change, and ultimately this will lead to improved health outcomes [70].

There are several strengths to this trial; for example, our trial will have more participants and measure intervention outcomes over a longer period than most comparable interventions in this space [18–21]. Additionally, our trial applies a robust study design, rigorous participant screening, device-measured physical activity outcomes, numerous measures to assess secondary outcomes, standardised exposure and contact across groups, and sufficient analytical power to observe between group changes. The lack of face-to-face contact with participants aligns with the nature of real-world digital interventions and allows the recruitment of participants anywhere in Australia, which enhances the generalisability of the study findings. However, a potential downside of this is a lower feeling of accountability towards study participation and higher levels of attrition. Nevertheless, this study and the intervention it aims to evaluate are well-positioned to generate new knowledge and advance the field in this space.

## Trial status

Protocol version 1.0, September 2024. Currently, the trial is ongoing and recruitment of participants continues. Recruitment began on 5 August 2024 and was expected to be completed by late October 2024.

Protocol version 2.0, February 2025. Currently, the trial is ongoing and recruitment was completed on 25 November 2024.

Protocol version 3.0, June 2025. Currently, the trial is ongoing for a small number of participants, i.e. those that were recruited early on have already completed their participation.

## Supplementary Information

Additional file 1: SPIRIT checklist.

## Data Availability

Upon study completion, only named study investigators will have access to the final trial dataset. While the data will not be publicly available, reasonable requests for access will be considered by the lead investigator (CV) in line with (additional) ethical approvals.
